# Dendritic Cell IL-1α and IL-1β Are Polyubiquitinated and Degraded by the Proteasome*

**DOI:** 10.1074/jbc.M114.595686

**Published:** 2014-11-04

**Authors:** Joseph S. Ainscough, G. Frank Gerberick, Maryam Zahedi-Nejad, Gloria Lopez-Castejon, David Brough, Ian Kimber, Rebecca J. Dearman

**Affiliations:** From the ‡ Faculty of Life Sciences, University of Manchester, Manchester M13 9PT, United Kingdom and; the § Procter & Gamble Company Co., Cincinnati, Ohio 45253

**Keywords:** Dendritic Cell, Inflammation, Interleukin 1 (IL-1), Proteasome, Ubiquitylation (Ubiquitination), IL-1α IL-1β

## Abstract

IL-1α and β are key players in the innate immune system. The secretion of these cytokines by dendritic cells (DC) is integral to the development of proinflammatory responses. These cytokines are not secreted via the classical secretory pathway. Instead, 2 independent processes are required; an initial signal to induce up-regulation of the precursor pro-IL-1α and -β, and a second signal to drive cleavage and consequent secretion. Pro-IL-1α and -β are both cytosolic and thus, are potentially subject to post-translational modifications. These modifications may, in turn, have a functional outcome in the context of IL-1α and -β secretion and hence inflammation. We report here that IL-1α and -β were degraded intracellularly in murine bone marrow-derived DC and that this degradation was dependent on active cellular processes. In addition, we demonstrate that degradation was ablated when the proteasome was inhibited, whereas autophagy did not appear to play a major role. Furthermore, inhibition of the proteasome led to an accumulation of polyubiquitinated IL-1α and -β, indicating that IL-1α and -β were polyubiquitinated prior to proteasomal degradation. Finally, our investigations suggest that polyubiquitination and proteasomal degradation are not continuous processes but instead are up-regulated following DC activation. Overall, these data highlight that IL-1α and -β polyubiquitination and proteasomal degradation are central mechanisms in the regulation of intracellular IL-1 levels in DC.

## Teaser

**Background:** Interleukin-1 secretion is an important process in inflammation and thus, the intracellular regulation of these cytokines is of interest.

**Results:** Inhibition of the proteasome in dendritic cells inhibits interleukin-1 degradation and leads to an accumulation of polyubiquitinated interleukin-1.

**Conclusion:** Interleukin-1 cytokines are regulated by polyubiquitination and proteasomal degradation.

**Significance:** Polyubiquitination and degradation are important processes in the intracellular regulation of interleukin-1.

## Introduction

Dendritic cells (DC)^2^ are of fundamental importance to the immune system, playing pivotal roles in the initiation and orchestration of immune responses (1, 2). They serve as dynamic antigen presenting cells that bridge the innate and adaptive immune systems. Thus, DC survey the local microenvironment, discriminating between a broad range of pathogenic and non-pathogenic cues and initiating immune responses, including inflammation (3). Inflammation is a complex response of the innate immune system that is associated with 5 characteristic features: erythema, edema, heat, pain, and loss of function (4, 5). These symptoms, which are crucial for the resolution of infection and injury, occur as a result of a series of changes driven by the production of proinflammatory cytokines, including members of the interleukin-1 (IL-1) family. IL-1α and IL-1β are closely related potent proinflammatory members of the IL-1 family and are produced by DC (6). The secretion of these cytokines is an integral component of the role of DC in orchestrating immune and inflammatory responses. Therefore, the transcriptional and post-transcriptional regulation of IL-1 by DC is of considerable importance, not only in the context of the resolution of infection and injury, but also in the context of preventing inappropriate or excessive inflammatory reactions. This is evident in pathologies such as gout (7), rheumatoid arthritis (8), cancer (9), and dementia (10), where the dysregulation of IL-1 is implicated strongly.

Unlike most cytokines, IL-1α and IL-1β are not secreted via the classical secretory pathway. Instead, IL-1 is secreted via a non-conventional pathway and its release requires two independent signals. The first signal is typically provided by pathogen-associated molecular patterns that act via pattern recognition receptors, such as members of the Toll-like receptor (TLR) family, to stimulate complex signaling pathways (11, 12). Ultimately, the activation of these pathways results in the translocation of the transcription factor, nuclear factor κ-light chain enhancer of activated B cells (NF-κB), to the nucleus. This drives the transcription of a variety of pro-inflammatory proteins, including IL-1α, IL-1β, and IL-6 (13), with IL-1α and IL-1β being transcribed as 31-kDa precursors (pro-IL-1). The secretion of these cytokines requires a second signal, which is provided usually by additional pathogen-associated molecular patterns or molecules associated with tissue trauma or damage (damage-associated molecular patterns). These damage-associated molecular patterns signal via cytosolic pattern recognition receptors, typically of the NOD-like receptor family, to stimulate assembly of inflammasome complexes (14). Formation of the inflammasome complex induces activation of the enzyme caspase-1 (15). Active caspase-1 cleaves pro-IL-1β to the 17-kDa bioactive form, facilitating its secretion (16). The inflammasome is also thought to be involved in IL-1α secretion, although this process is less well characterized and is also dependent on the calcium-dependent protease calpain. Similarly, the processing and secretion of pro-IL-1α involves cleavage into its 17-kDa form (17, 18).

Although many studies have focused on the regulation of IL-1 secretion, the intracellular control of pro-IL-1 remains poorly understood. Both pro-IL-1α and pro-IL-1β are cytosolic and may therefore be subject to a number of post-translational modifications. Such modifications represent an important mechanism by which cells can regulate the characteristics and function of proteins. For example, post-translational modifications have been shown to act as regulators at various stages of the NF-κB signaling pathway (19). Ubiquitination involves the addition of ubiquitin, an 8.5-kDa protein, to a given substrate (20). This process is mediated by a series of enzymes: E1, E2 and E3, that act sequentially to bind ubiquitin covalently to a Lys residue on the substrate protein. It is the final E3 ubiquitin ligase that confers the substrate specificity for ubiquitination. Substrate proteins may remain monoubiquitinated or may have further ubiquitin molecules added (polyubiquitination). In the formation of polyubiquitin chains, the carboxyl group on ubiquitin can bind to a number of different residues on the following ubiquitin, thus giving rise to various forms of polyubiquitination. Ultimately, the type of ubiquitin chain bound has a fundamental impact on the functional outcome of the modification (21). As an example, Lys^48^-linked polyubiquitin chains serve to target proteins for proteasomal degradation (22), whereas Lys^63^-linked polyubiquitin chains function in the regulation of the NF-κB pathway (23).

Here, we provide evidence that in murine DC, IL-1α, and IL-1β are polyubiquitinated and that, in both DC and macrophages, this polyubiquitination drives the proteasomal degradation of IL-1. Furthermore, these data demonstrate that in the presence of a second signal, polyubiquitinated IL-1 is still available for secretion. Collectively, our results demonstrate that in DC, the polyubiquitination and proteasomal degradation of IL-1 serves as an essential process in the regulation of IL-1 and, therefore, should be considered as an extra dimension to the current two-signal paradigm of IL-1 release.

## EXPERIMENTAL PROCEDURES

### 

#### 

##### Animals

Female BALB/c mice (6–8 weeks old) were used throughout these experiments (Harlan Olac, Bicester, UK). Mice were provided with environmental stimuli (bedding and nesting materials), food (SDS PCD pelleted diet; Special Diets Services Ltd, Witham, UK) and water were available *ad libitum*. Relative humidity was 55 ± 10% with a 12-h light/dark cycle and ambient temperature was maintained at 21 ± 2 °C. Maintenance and treatment of animals were conducted as specified by the United Kingdom Animals (Scientific Procedures) Act 1986. Mice were sacrificed by exposure to CO_2_ gas in rising concentrations followed by dislocation of the neck in concordance with schedule 1 (U.K. Animals (Scientific Procedures) Act 1986).

##### Antibodies and Reagents

LPS from *Escherichia coli* serotype 055:B5 (TLR2/4), poly(I:C), ATP, the autophagy inhibitor wortmanin and the translation inhibitor cycloheximide (CHX) were purchased from Sigma. The proteasome inhibitor MG132 was obtained from Merck Millipore (Billerica, MA). Recombinant murine pro-IL-1β was purchased from Affymetrix eBioscience (San Diego, CA). For Western blot analysis, the primary antibodies were goat anti-mouse IL-1α antibody, goat anti-mouse IL-1β antibody (both R&D Systems; Minneapolis, MN), or mouse anti-ubiquitin antibody (Santa Cruz Biotechnology, Santa Cruz, CA). The HRP-conjugated secondary antibodies were rabbit anti-goat IgG antibody (DAKO, Copenhagen, Denmark) and goat anti-mouse light chain antibody (Millipore).

##### Generation and Culture of Murine Bone Marrow-derived DC

Murine bone marrow-derived (BM) DC were generated following a previously described method (24). Briefly, bone marrow was extracted by flushing the tibias and femurs with PBS. The cell suspension was centrifuged at 200 × *g* for 5 min at room temperature. The remaining pellet was resuspended in pre-warmed, FCS-supplemented culture medium (RPMI 1640; Invitrogen), containing 400 μg/ml of penicillin/streptomycin, 292 μg/ml of l-glutamine, 0.05 mm 2-mercaptoethanol, 4 ng/ml of GM-CSF (Miltenyi Biotech, Bisley, UK), and 10% FCS (Invitrogen). A viable cell count was performed by trypan blue exclusion (0.5%; Sigma). Cells were cultured at ∼2 × 10^6^ cells/ml in Petri dishes and incubated at 37 °C. The cultures were fed on day 3 by addition of 10 ml of fresh culture medium, and again on day 6 by gentle aspiration of 10 ml of medium followed by the addition of 10 ml of fresh culture medium.

##### BMDC Treatments

BMDC were plated on day 8, in culture medium without GM-CSF, at 10^6^ cells/well (24-well plate) or 10^7^ cells/well (6-well plate; 10^6^ cells/ml). Following an initial 24-h dose-response experiment to determine the optimum dose of LPS to induce IL-1 production, cells were primed using 0.1 μg/ml of LPS. BMDC were primed with LPS as indicated in the text, and were activated with various concentrations of ATP for 30 min at the end of the culture. MG132, wortmanin, or a DMSO control were added for the final 4 h of incubation. CHX was added for the final 1 h of incubation. After incubation, supernatants were harvested and frozen at −80 °C. Cell lysates were harvested in 200 μl of lysis buffer (20 mm Tris-HCl, 137 mm NaCl, 20 mm EDTA, 10% glycerol, 0.5% Ipegal, 1 mm PMSF, protease inhibitor mixture (1:100)) and frozen at −80 °C. For PCR analysis, lysates were prepared for RNA extraction following the manufacturer's instructions (Purelink RNA mini kit; Invitrogen).

##### Immunoprecipitation of IL-1

To prepare lysates for immunoprecipitation, supernatants were removed and cells were washed twice with PBS. Cells were incubated on ice with wash buffer (20 mm *N*-ethylmaleimide in PBS). After a final wash with PBS, cell lysates were prepared in 500 μl of a specialized lysis buffer, formulated to prevent deubiquitination (25 mm Tris, pH 7.4, 150 mm NaCl, 0.5% sodium deoxycholate, 1% Triton X-100, 0.1% sodium dodecyl sulfate, 5 mm *N*-ethylmaleimide, 1 mm PMSF). An aliquot of lysate (50 μl) was retained as whole cell lysate (WCL) and the remainder was immunoprecipitated using an anti-IL-1α or anti-IL-1β antibody (both antibodies were capture antibodies supplied in the R&D ELISA Duosets). Briefly, samples were incubated overnight with 2 μg of antibody at 4 °C. Protein G-Sepharose beads (50 μl; Sigma) were added to each sample for 2 h at 4 °C. After incubation, the samples were washed three times by centrifugation at 10,000 × *g* for 30 s and supernatants were removed. The Sepharose beads were then resuspended in 1 ml of lysis buffer. After the final wash, the beads were resuspended in 50 μl of 2 × sample buffer (Bio-Rad) containing 1% 2-mercaptoethanol. Immunoprecipitated protein was eluted from the beads following heat treatment (80 °C for 5 min).

##### ELISA

Supernatants and lysates were analyzed for IL-1α or IL-1β protein using specific ELISA Duosets from R&D Systems. ELISA were performed following the manufacturer's instructions. An IL-6 ELISA was performed as described previously (25). The lower limits of accurate detection for IL-1 and IL-6 were ∼62 and 156 pg/ml, respectively.

##### Western Blots

In preparation for Western blot analysis, supernatants and lysates were diluted in sample buffer (Bio-Rad) containing 1% 2-mercaptoethanol and heated at 80 °C for 5 min. Samples were resolved on a 10% acrylamide gel and proteins were transferred to a nitrocellulose membrane. Specific proteins were detected using anti-IL-1α, anti-IL-1β (both 0.1 μg/ml), or anti-ubiquitin antibodies (0.2 μg/ml). Subsequently, blots were incubated with either HRP-labeled anti-IgG antibody (for IL-1α or IL-1β; 0.25 μg/ml) or HRP-labeled anti-light chain IgG antibody (ubiquitin; 0.25 μg/ml). Proteins were visualized using enhanced chemiluminescence reagents (Thermo Scientific, Waltham, MA).

##### RT-PCR

Total RNA was purified from samples using Purelink RNA mini kit and converted to cDNA using a high capacity RNA to cDNA kit (Invitrogen). mRNA expression levels of mouse IL-1β were determined by RT-PCR using a TaqMan primer obtained from Invitrogen using a RT-PCR machine (StepOne plus). Expression was normalized using untreated cells (control) and to hypoxanthine-guanine phosphoribosyltransferase, with the ΔΔ*C_t_* method used to calculate the relative fold-change.

##### Statistical Analysis

Statistical analysis was performed using GraphPad Prism 6 software. Data were analyzed by one-way ANOVA to determine overall differences and a Tukey post-hoc test was performed to determine statistically significant differences between treatment groups; *, *p* < 0.05; **, *p* < 0.01.

## RESULTS

### 

#### 

##### Pro-IL-1α and Pro-IL-1β Are Degraded by an Active Cellular Process

**FIGURE 1.**
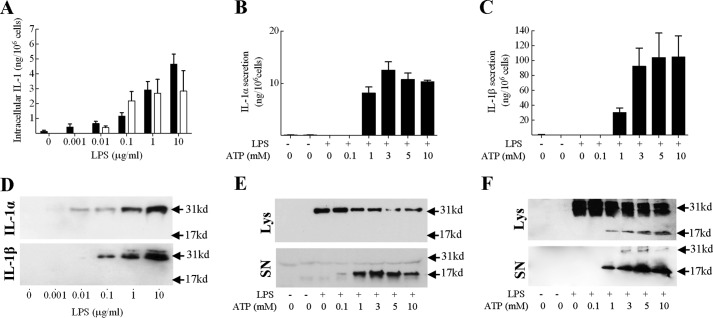
**Intracellular expression and secretion of IL-1 by BMDC: impact of LPS and ATP.** BMDC (10^6^ cells/ml) were cultured for 24 h in the presence of medium alone or increasing doses of LPS (*A* and *D*), or for 4 h with medium alone or LPS (0.1 μg/ml) and then challenged with ATP (0–10 mm) for 30 min (*B*, *C*, *E*, and *F*). Supernatants and cell lysates were harvested and analyzed for the presence of IL-1α or IL-1β using cytokine-specific ELISA. IL-1 content of cell lysates is displayed in *A* (■, IL-1α; □, IL-1β) and supernatants (secreted IL-1) in *B* (IL-1α) and *C* (IL-1β) as mean ± S.E. (*n* = 3). Supernatants (*SN*) and lysates (*Lys*) were also analyzed by Western blotting using either anti-IL-1α (*D* and *E*) or anti-IL-1β (*D* and *F*) antibodies. A protein marker lane on each gel was used to determine molecular weight. Representative blots are shown in each case.

In initial experiments, it was confirmed that the regulation of IL-1 production in DC was consistent with the current paradigm of IL-1 secretion (26), requiring two signals. Stimulation of BMDC with the TLR 4 ligand LPS resulted in an up-regulation in intracellular (lysate) expression of both pro-IL-1α and pro-IL-1β protein, without inducing detectable secretion (Fig. 1*A*). The intracellular IL-1 induced by LPS was exclusively 31-kDa in size (corresponding to the IL-1 precursor, pro-IL-1) as determined by Western blot analysis (Fig. 1*D*). In subsequent experiments signal 2 was provided, by ATP. ATP acts via the P2X7 receptor to induce activation of the NLRP3 inflammasome (27–29) and thus the cleavage and secretion of IL-1. Here, challenge of LPS-primed BMDC with ATP (1 to 10 mm) resulted in IL-1α (Fig. 1, *B* and *E*) and IL-1β (Fig. 1, *C* and *F*) processing and release.

One interesting observation made was that the total amount of IL-1β appeared to increase dramatically following optimal ATP challenge (from ∼5 to 100 ng/10^6^ cells). Given that the ATP is only added for the final 30 min of incubation, it seemed unlikely that this increase reflected an actual increase in protein expression. Indeed, under these conditions IL-1β mRNA levels were unaffected by ATP stimulation (data not shown). Consequently, it was hypothesized that the IL-1β ELISA used has a greater avidity for the mature cytokine, relative to the 31-kDa precursor. To investigate this, recombinant pro-IL-1β and the recombinant mature IL-1β were analyzed in parallel in the ELISA over a concentration range of 300 to 1.2 pm. It was demonstrated that the ELISA used does indeed have a considerably greater avidity for the mature cytokine, relative to the 31-kDa precursor (data not shown).

**FIGURE 2.**
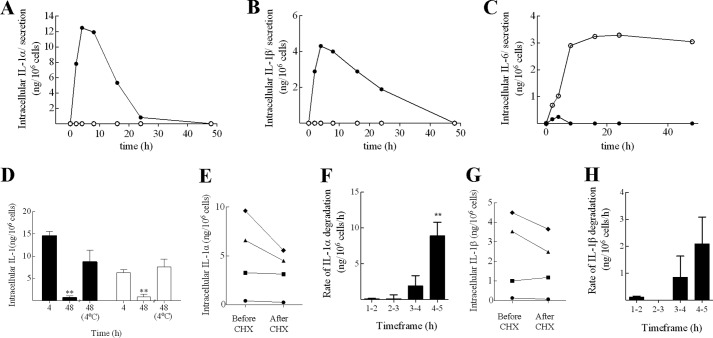
**IL-1 is degraded intracellularly in BMDC by a process up-regulated during DC activation.** BMDC (10^6^ cells/ml) were incubated with LPS (0.1 μg/ml) for various time periods (0 to 48 h). Supernatants (○) and lysates (●) were analyzed for the presence of IL-1α (*A*), IL-1β (*B*), and IL-6 (*C*) using specific ELISA (single experiment). In some experiments, cells were incubated with LPS for 4 or 48 h, with the final 44 h at 37 or 4 °C, and IL-1α (■) and IL-1β (□) content was measured in lysates by ELISA (*D*; *n* = 3). In other experiments, cells were incubated with LPS for various periods of time (1 to 5 h), with the final 1 h of culture in the presence of CHX (10 μg/ml)(*E–H*). Lysates were prepared and analyzed for the presence of IL-1α (*E* and *F*) and IL-1β (*G* and *H*) by ELISA. Data are displayed from one representative experiment with respect to actual cytokine levels for each time period before and after a 1-h CHX treatment (●, 1–2 h; ■, 2–3 h; ▴, 3–4 h; ♦, 4–5 h) (*E,* IL-1α; and *G*, IL-1β). The rate of IL-1 degradation for each time period (ng/10^6^ cells/h) was calculated by deducting cytokine levels after the CHX incubation from baseline levels prior to CHX addition (*F,* IL-1α; and *H,* IL-1β). Data shown are mean ± S.E. (*n* = 3). Statistical significance of differences between 4 (*D*) or 1–2 h-treated samples *versus* other treatment groups (*F* and *H*) was determined by one-way ANOVA, *, *p* < 0.05; **, *p* < 0.01.

To investigate the intracellular regulation of pro-IL-1α and -β in BMDC, the kinetics of IL-1 protein expression in response to LPS alone was examined. Here, stimulation with LPS caused an up-regulation of both pro-IL-1 forms (Fig. 2, *A* and *B*). The expression of both IL-1α and IL-1β were transient, peaking at 4 h post-stimulation and decreasing thereafter. In contrast, the classically secreted cytokine IL-6 (30) was secreted almost as soon as it was produced and accumulated in the supernatant (Fig. 2*C*). Despite the reduction in intracellular cytokine, IL-1 secretion was not detected at any time point, indicating that both IL-1α and IL-1β were degraded intracellularly. Repeat experiments confirmed that the loss of IL-1 between 4 and 48 h was statistically significant (Fig. 2*D*; *p* < 0.01). Furthermore, the process was temperature-dependent, such that incubation of LPS-primed BMDC at 4 °C, rather than 37 °C, largely abrogated the effect (Fig. 2*D*). As incubation at 4 °C effectively inhibits cellular metabolic activity, these data show that IL-1 degradation is dependent on an active, cellular process.

##### IL-1 Degradation Is Initiated 4 h after LPS Stimulation

To explore the nature of IL-1 degradation further, the early kinetics (1–5 h) of LPS-induced IL-1α and IL-1β degradation were investigated in more detail. To remove the potentially confounding influence of *de novo* protein production, the translation inhibitor CHX was utilized. Specifically, degradation was measured over 1-h intervals (1–2, 2–3, 3–4, and 4–5 h) by measurement of IL-1 levels before and after a 1-h pulse with CHX. Raw data from one representative experiment are presented in Fig. 2, *E* and *G*, illustrating cytokine levels before and after CHX treatment for each time interval. IL-1 degradation was not apparent during the 1–2 or 2–3 h time frames (no decrease in IL-1 levels), whereas marked losses in cytokine were recorded during the 3–4 and 4–5 h time frames (before and after CHX treatment). Subsequently, the rate of degradation during each interval was calculated (Fig. 2, *F* and *H*; 3 independent experiments). For IL-1α, the rate of degradation was negligible in the first 3-h post-LPS stimulation, but increased rapidly thereafter, reaching ∼10 ng/10^6^ cells/h at 4–5 h. A similar pattern was recorded for IL-1β, with the rate of degradation reaching 2 ng/10^6^ cells/h at 4–5 h. Overall, these results demonstrate that after a lag of 2–3 h following LPS stimulation, the degradation of IL-1 is induced.

##### IL-1 Degradation Is Inhibited by the Addition of the Proteasome Inhibitor MG132

**FIGURE 3.**
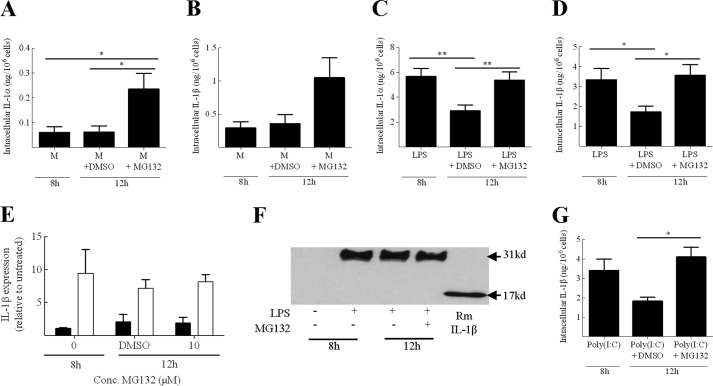
**IL-1 degradation in BMDC is dependent upon the proteasome.** BMDC (10^6^ cells/ml) were incubated with medium alone (M; *A* and *B*) or LPS (0.1 μg/ml; *C* and *D*) or poly(I:C) (100 μg/ml; *G*) for 8 or 12 h with the final 4 h in the presence of 10 μm of the proteasome inhibitor MG132 (*A–D* and *G*) or with an equivalent volume of solvent (DMSO) alone. Supernatants and lysates were prepared and analyzed for the presence of IL-1α (*A* and *C*) and IL-1β (*B* and *D*) using cytokine-specific ELISA. For both IL-1α and IL-1β, secreted (supernatant) cytokine levels were below the limit of detection (data not shown). Lysates prepared in parallel (■, medium; □, LPS) were also analyzed for IL-1β mRNA using RT-PCR and the ΔΔ*C_t_* method. Results were normalized against naive BMDC and the housekeeping gene hypoxanthine-guanine phosphoribosyltransferase (HPRT) (*E*). These lysates (and recombinant (*Rm*) IL-1β control) were also analyzed by Western blotting using an anti-IL-1β (*F*) antibody. A representative blot is shown. A protein marker lane on the gel was used to determine molecular mass. Data shown are mean ± S.E. (*n* = 4). A one-way ANOVA was used to determine statistical significance of differences between treatment groups. *, *p* < 0.05; **, *p* < 0.01.

Next we examined the mechanism of cytokine degradation in DC in more detail. In a previous study, Moors *et al.* (31) suggested that in human monocytes, IL-1β degradation is mediated by the proteasome. However, in a conflicting study using mouse macrophages and DC, Harris *et al.* (32) suggested that IL-1β degradation is mediated by autophagy. The degradation of IL-1α has not been explored previously. In the current investigations, DC were primed for 8 h with LPS to up-regulate intracellular IL-1 expression. To characterize the process of IL-1 degradation, BMDC were then incubated for a further 4 h in the presence or absence of the proteasome inhibitor MG132 or the autophagy inhibitor wortmanin. The marked degradation of IL-1α and IL-1β in control (DMSO-treated) LPS-primed BMDC was completely abrogated by addition of 10 μm MG132 (Fig. 3, *C* and *D*). Interestingly, the addition of MG132 to unprimed cells, which have a relatively low baseline expression of IL-1, caused a marked increase in both IL-1 cytokines. This increase was statistically significant for IL-1α (*p* < 0.05), suggesting that the proteasome acts to regulate basal and LPS-induced IL-1 turnover (Fig. 3, *A* and *B*).

Previous studies have suggested that the proteasome may be involved in regulating the NF-κB signaling pathway (33, 34) and the inflammasome (35). Therefore, the stabilization of IL-1 levels after inhibition of the proteasome observed here may reflect increased IL-1 expression or processing rather than an inhibition of IL-1 degradation. However, analysis of IL-1β mRNA expression showed that the addition of MG132 to both untreated or LPS-primed BMDC was without effect on IL-1β mRNA levels (Fig. 3*E*). Western blot analysis confirmed that the IL-1 was the 31-kDa precursor form regardless of addition of the proteosomal inhibitor (Fig. 3*F*). Thus, MG132 did not affect IL-1 processing or transcription.

Harris *et al.* (32) suggested that the sequestration of IL-1β into autophagosomes was dependent on the TRIF signaling pathway. As LPS signals via both TRIF and MyD88 signaling pathways, it was important to explore IL-1β degradation where the IL-1 expression was up-regulated via the TRIF signaling pathway only. TLR3 has been shown to signal via the TRIF signaling pathway exclusively and so the TLR3 ligand poly(I:C) was used to investigate degradation here. Once again, there was a marked degradation of IL-1β between 8 and 12 h (Fig. 3*G*). Importantly, this degradation was completely inhibited by proteasome inhibition, suggesting that IL-1 degradation under these conditions is proteasomal, even when the up-regulation is dependent on the TRIF signaling pathway.

**FIGURE 4.**
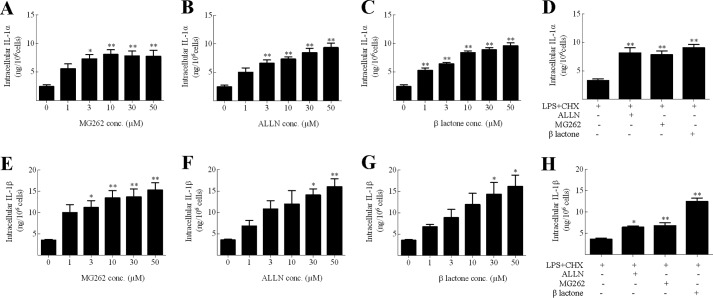
**IL-1 degradation is dependent on the proteasome in macrophages.** J774 (*A–C* and *E–G*) or BMDM (*D* and *H*) (10^6^ cells/ml) were incubated with LPS (1 μg/ml) for 16 h with a final 4 h in the presence of CHX (10 μg/ml) and DMSO, MG262 (*A* and *E*), ALLN (*B* and *F*), or β lactone (*C* and *G*) (all 0–50 μm for J775 cells). For BM-derived macrophages a single concentration of each proteasome inhibitor was used (50 μm for ALLN; 30 μm for both MG262 and β lactone). Supernatants and lysates were prepared and analyzed for the presence of IL-1α (*A–D*) and IL-1β (*E–H*) using cytokine-specific ELISA. For both IL-1α and IL-1β, secreted cytokine levels were below the limit of detection (data not shown). Data shown are mean ± S.E. (*n* = 3). A one-way ANOVA was used to determine statistical significance of differences between the DMSO-treated samples *versus* the other treatment groups. *, *p* < 0.05; **, *p* < 0.01.

To examine whether proteosomal degradation was a general feature of IL-1 production in cell types other than DC, parallel experiments were conducted using murine BM-derived macrophages and the murine macrophage cell line, J774 (Fig. 4). Cells were primed with LPS for 16 h, with the last 4 h being in the presence of CHX (to inhibit further IL-1 translation) alone or in the presence of various proteosomal inhibitors: MG262 (an inhibitor from the same chemical series as MG132 (37)), ALLN or β lactone (all at 1–50 μm formulated in DMSO, or DMSO alone control) and IL-1α or IL-1β expression was measured. There was no detectable secretion of cytokine, but LPS priming of both primary macrophages and the cell line resulted in intracellular cytokine expression (∼2.5 ng/10^6^ cells). For J774 cells, a dose-dependent increase in cytokine content was recorded in the presence of each of the three proteosomal inhibitors. Similar increases in IL-1 were recorded at optimal doses (30–50 μm) of all three inhibitors in parallel experiments conducted with BM-derived macrophages. Thus, IL-1 degradation in murine macrophages is also dependent upon the proteasome.

**FIGURE 5.**
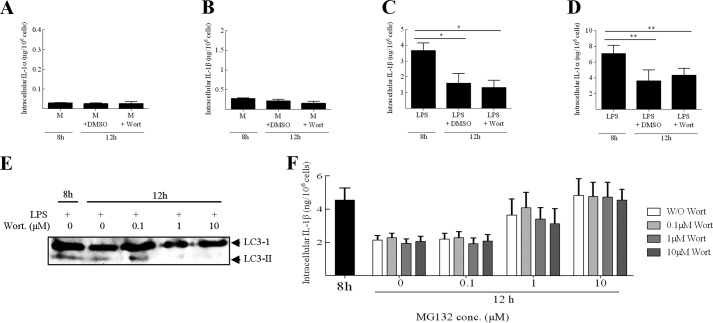
**IL-1 degradation in BMDC is not dependent upon autophagy.** BMDC (10^6^ cells/ml) were incubated with medium alone (*M*; *A* and *B*) or LPS (0.1 μg/ml; *C*, *D*, and *F*) for 8 h or for 12 h with the final 4 h in the presence of 10 μm of the autophagy inhibitor wortmanin (*Wort*) (*A-D*) or with an equivalent volume of solvent (DMSO) alone, or both wortmanin and MG132 in various combinations of concentrations (*F*; 0.1, 1, or 10 μm). Supernatants and lysates were prepared and analyzed for the presence of IL-1α (*A* and *C*) and IL-1β (*B*, *D*, and *F*) using cytokine-specific ELISA. For both IL-1α and IL-1β, secreted (supernatant) cytokine levels were below the limit of detection (data not shown). In addition, BMDC (10^6^ cells/ml) were incubated with LPS (0.1 μg/ml) for 8 h, or 12 h with the final 4 h in the presence or absence of wortmanin (0.1, 1, or 10 μm). Lysates were prepared and analyzed by Western blotting using an anti-LC3 antibody (*E*). A protein marker lane on the gel was used to determine molecular weight. Data shown are mean ± S.E. (*n* = 4). A one-way ANOVA was used to determine statistical significance of differences between treatment groups. *, *p* < 0.05; **, *p* < 0.01.

To investigate whether autophagy was implicated in IL-1 degradation, it was first confirmed that under the conditions utilized, wortmanin was indeed an autophagy inhibitor in BMDC. The addition of wortmanin to LPS-primed cells resulted in a dose-dependent reduction in the conversion of LC3-I to LC3-II with complete inhibition observed at 10 μm (Fig. 5*E*) (36). Importantly, the addition of wortmanin had no effect on cell viability (data not shown), confirming that the inhibition of LC3 conversion was indeed due to an inhibition of autophagy and not an artifact as a result of cytotoxicity. Here, the addition of 10 μm wortmanin had no impact on the degradation of IL-1α and IL-1β in LPS-primed BMDC (Fig. 5, *C* and *D*). The addition of 10 μm wortmanin also had no effect on the basal turnover of IL-1 (Fig. 5, *A* and *B*), however, it must be emphasized that the inhibition of autophagy by wortmanin was not confirmed in unprimed cells and so a role for autophagy under these conditions cannot be ruled out completely.

To test whether autophagic and proteasomal pathways were synergistic in IL-1 degradation, we explored degradation under conditions of both proteasome and autophagy inhibition (Fig. 5*F*). However, these data indicate that IL-1 degradation under these conditions was unaffected by autophagy inhibition, even under conditions of partial (1 μm MG132) or total proteasome inhibition (10 μm MG132). Together, these data suggest that degradation of both IL-1α and IL-1β over the period studied here was dependent upon the proteasome in DC and macrophages.

##### Intracellular IL-1 Is Polyubiquitinated

**FIGURE 6.**
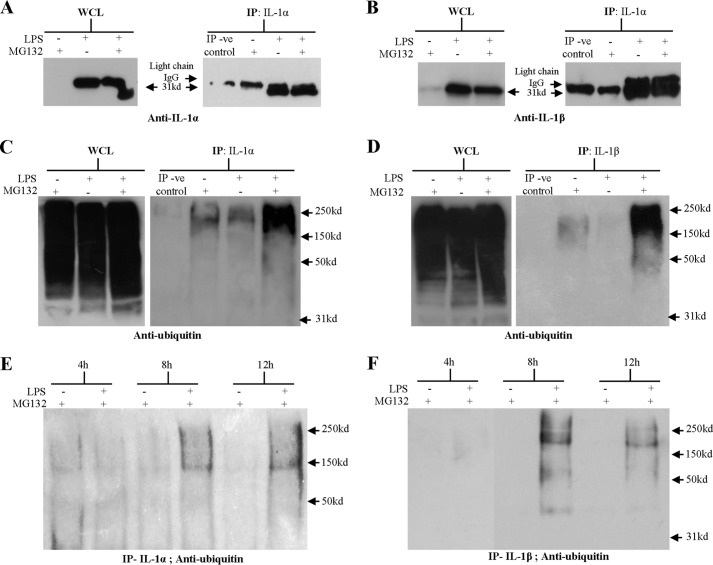
**IL-1 is polyubiquitinated in DC.** 10^7^ BMDC (10^6^ cells/ml) were incubated with medium or LPS (0.1 μg/ml) for 12 h with the final 4 h in the presence or absence of 10 μm of the proteasome inhibitor MG132 (*A–D*). In addition, 10^7^ BMDC (10^6^ cells/ml) were incubated with medium or LPS (0.1 μg/ml) for 4, 8, or 12 h with the final 4 h in the presence of 10 μm of the proteasome inhibitor MG132 (*E* and *F*). Cells were lysed, an aliquot of lysate was retained as whole cell lysate (*WCL*) and the remainder was immunoprecipitated with anti-IL-1α (*A*, *C*, and *E*) or anti-IL-1β antibody (*B*, *D*, and *F*). The same volume of lysis buffer alone was immunoprecipitated (*IP*) with anti-IL-1α or anti-IL-1β antibody (immunoprecipitation negative control; *IP-ve control*). The samples were analyzed by Western blotting using an anti-IL-1α antibody (*A*), an anti-IL-1β antibody (*B*), or an anti-ubiquitin antibody (*C–F*). For Fig. 4*D*, results are from 2 separate gels, run and developed concurrently. A protein marker lane on each gel was used to determine the molecular weight. Representative blots are shown in each case.

To explore whether IL-1α and IL-1β conform to the classic paradigm of proteasomal degradation, the ubiquitination status of IL-1 was investigated. Here, stimulation of BMDC with LPS, or LPS in the presence of MG132 to block degradation, resulted in a strong pro-IL-1α and pro-IL-1β signal, as determined by Western blot analysis of the WCL (Fig. 6, *A* and *B*). Pro-IL-1α and IL-1β were successfully immunoprecipitated from these LPS-primed BMDC lysates using appropriate antibodies, again as determined by Western blotting. In both the anti-IL-1α and anti-IL-1β-immunoprecipitated fractions, a band of ∼25 kDa was observed in control lanes (unprimed BMDC lysates and lysis buffer-negative control). The size of this band is consistent with light chain IgG antibody fragments and is therefore likely to be due cross-reactivity between the immunoprecipitation antibodies and the anti-IgG secondary antibody used for detection.

An anti-ubiquitin Western blot of the WCL confirmed that each BMDC sample contained many ubiquitinated proteins, ranging from 30 to 250 kDa in size (Fig. 5, *C* and *D*). The immunoprecipitated IL-1α and IL-1β from LPS-primed, MG132-treated BMDC were also found to contain large amounts of ubiquitinated protein, representing an accumulation of ubiquitinated IL-1. Ubiquitinated IL-1 ranged in size from 40 to 250 kDa. Given that ubiquitin is only 8.5 kDa (38), the size of the ubiquitinated IL-1 indicates that both IL-1α and IL-1β are polyubiquitinated. A much weaker smear of ubiquitinated IL-1 was also detected in the immunoprecipitated IL-1 from unprimed MG132-treated samples, supporting previous evidence that proteasomal degradation also regulates the basal turnover of IL-1. Interestingly, in LPS-primed BMDC in the absence of proteosomal inhibition, a weak smear of ubiquitinated IL-1 was also detected in the immunoprecipitated IL-1α but not the IL-1β. To provide further support that the process of IL-1 degradation is initiated some 4 h after LPS stimulation, the kinetics of IL-1 ubiquitination were investigated. BMDC were incubated with medium or LPS and incubated for 4, 8, or 12 h, with the final 4 h in the presence MG132 (Fig. 6, *E* and *F*). Here, an anti-ubiquitin Western blot of the IL-1 immunoprecipitated from 4 h LPS-primed BMDC did not detect ubiquitinated protein, whereas samples from 8- or 12-h primed cells contained ubiquitinated IL-1.

##### Intracellular Polyubiquitinated IL-1 Is Lost following ATP Activation

**FIGURE 7.**
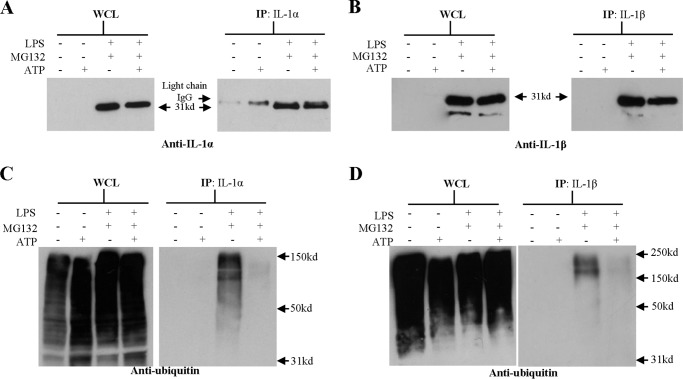
**Ubiquitinated IL-1 expression following LPS priming and ATP challenge.** 10^7^ BMDC (10^6^ cells/ml) were incubated with medium or LPS (0.1 μg/ml) for 12 h with the final 4 h in the presence or absence of 10 μm of the proteasome inhibitor MG132. Cells were then challenged with 10 mm ATP for 30 min or left untreated. Cells were lysed, an aliquot of lysate was retained as whole cell lysate (*WCL*) and the remainder was immunoprecipitated (*IP*) with anti-IL-1α (*A* and *C*) or anti-IL-1β antibody (*B* and *D*). The samples were analyzed by Western blotting using an anti-IL-1α antibody (*A*), an anti-IL-1β antibody (*B*), or an anti-ubiquitin antibody (*C* and *D*). A protein marker lane on each gel was used to determine molecular weight. Representative blots are shown in each case.

Having shown that IL-1α and IL-1β are polyubiquitinated in DC following signal 1, the impact of signal 2 (ATP) on ubiquitination was investigated. Here, Western blot analysis of the WCL showed that LPS-primed BMDC expressed large amounts of pro-IL-1, regardless of challenge with ATP (Fig. 7, *A* and *B*). As before, pro-IL-1α and pro-IL-1β were successfully immunoprecipitated from the LPS-primed BMDC lysates. Parallel analysis of the supernatants by ELISA confirmed that ATP induced the secretion of IL-1 from LPS-primed BMDC, despite the presence of MG132 (data not shown). Anti-ubiquitin Western blot analysis of the WCL revealed that all samples contained a range of different polyubiquitinated proteins (30–150 kDa). As previously shown, IL-1 immunoprecipitated from LPS-primed, MG132-treated samples contained large amounts of polyubiquitinated IL-1 (Fig. 7, *C* and *D*). In contrast, IL-1 immunoprecipitated from ATP stimulated, LPS-primed and MG132-treated BMDC contained very little polyubiquitinated IL-1 suggesting that ATP stimulation targeted polyubiquitinated IL-1 for secretion. Interestingly, an anti-ubiquitin Western blot of the supernatants revealed that there was no detectable ubiquitinated protein released from the ATP-stimulated, LPS-primed, and MG132-treated BMDC (data not shown), despite the detection of secreted IL-1 by ELISA, indicating that none of the secreted proteins, including IL-1, were ubiquitinated, at least within the limits of detection of the anti-ubiquitin antibody. Taken together, these results suggest that the sequence of events is that ubiquitinated pro-IL-1 is targeted for cleavage, and that the ubiquitin chain is removed prior to secretion, most likely in the same event that cleaves the pro-IL-1 into its mature form. However, Lopez-Castejon *et al.* (44) recently reported that deubiquitinases play a role in regulating IL-1 release by affecting inflammasome function. Therefore, the possibility that ATP influences, directly or indirectly, the deubiquitination of IL-1 cannot be discounted and thus, should be the subject of further investigation.

## DISCUSSION

The release of IL-1 from DC is an important step in the initiation of the inflammatory response (39). Consistent with previous publications (40), the current studies have shown that IL-1 secretion by DC requires two signals; one signal to increase precursor expression, and a second signal to stimulate caspase-1 activation and to facilitate cytokine release. Given the potency of these cytokines, the complexity of this mechanism reflects the need for tight control of IL-1α and IL-1β production. Here we report that both IL-1α and IL-1β are degraded by the proteasome and suggest that this degradation mechanism acts as an important regulator of intracellular IL-1.

The degradation of IL-1α had not been previously investigated, and the degradation of IL-1β is poorly understood. It has been suggested that in human monocytes, IL-1β degradation is mediated by the proteasome (31). However, a conflicting paper using human and mouse macrophages, as well as mouse DC, indicated that IL-1β degradation is controlled by autophagy (32). In the current investigations, the degradation of both IL-1α and IL-1β has been shown to be dependent on the proteasome in mouse DC. In addition, we provide evidence suggesting that IL-1 degradation in macrophages is also dependent on the proteasome.

Ubiquitination is rapidly emerging as a key player in the regulation of immune responses and thus the direct polyubiquitination of IL-1 as demonstrated herein is of great interest (43, 44). Ubiquitination is implicated as a fundamental regulator at various stages of the canonical NF-κB signaling pathway. Recent evidence has highlighted the importance of ubiquitination in this pathway by showing that the activation of the ubiquitin ligase TNF receptor-associated factor 6 induces the Lys^63^-linked polyubiquitin of NF-κB essential modulator and receptor-interacting protein 1. These Lys^63^-linked polyubiquitin chains then serve as a scaffold for the recruitment of a kinase complex, which, when assembled, drives the activation of NF-κB (23, 45). In addition, we recently reported that in macrophages, ubiquitination may also be implicated in the assembly of the inflammasome (44, 46). As the inhibition of the proteasome caused a marked increase in levels of polyubiquitinated IL-1, and as the classical role of ubiquitination is to target a protein for degradation (47), the probable role of IL-1 polyubiquitination is to target these cytokines for degradation. However, given that it is also known that ubiquitination is a diverse modification with a range of immunoregulatory functions, it is tempting to speculate that polyubiquitinated IL-1 may also have an immunoregulatory role, potentially modulating the pathways that drive IL-1 processing and secretion.

Having shown that IL-1 is polyubiquitinated and consequently degraded in DC, it is of interest to consider the function of this degradation *in vivo*. One obvious role for IL-1 degradation is to prevent the intracellular accumulation of pro-IL-1. The importance of this is clear when it is considered that the purpose of the 2-signal system of IL-1 release is to allow secretion only when a perceived threat is significant enough to warrant inflammation. A stimulus that induces IL-1 expression without concurrent inflammasome activation or vice versa is not likely to be sufficiently dangerous and thus, will not induce secretion in DC. However, without rapid degradation in DC, these cytokines would persist intracellularly as inactive precursors and therefore the need for 2 signals to induce IL-1 secretion would be ablated. Thus, relatively innocuous threats could drive unnecessary and potentially damaging inflammatory responses. Another role for IL-1 degradation may be in regulating the amount of IL-1 available for secretion. The current investigations demonstrate that the process of ubiquitination and proteasomal degradation is induced by DC activation and that basal turnover of IL-1 is regulated by the proteasome. Together, these data suggest that there is a basal level of ubiquitination and that DC activation drives the up-regulation or the activation of proteins that mediate IL-1 ubiquitination. As the E3 ubiquitin ligases are relatively specific for the protein that they ubiquitinate (48), the hypothesis is that it is the E3 ubiquitin ligase of IL-1 that is up-regulated or activated as a consequence of DC priming. If this is true, expression levels of the E3 ubiquitin ligase of IL-1 may be modulated to control the amount of IL-1 in the cell at any one time. This regulation may play a crucial role *in vivo*, acting to constrain the amount of IL-1 available for secretion, both in the resting state and during the inflammatory response. This is, however, speculative and thus requires further investigation.

As previously discussed, the dysregulation of IL-1 is implicated in a number of debilitating diseases. In Alzheimer disease, IL-1 has been shown to be markedly overproduced in both experimental animal models such as the rat (10) and in humans (49, 50). Likewise, IL-1 has also been shown to be elevated in the cerebrospinal fluid of patients with Parkinson disease (51). IL-1 also exacerbates acute brain injury such as stroke (52) and thus, its dysregulation is of significance clinically. Although data exist to suggest the overproduction of IL-1 in these diseases is associated with an increase in IL-1 mRNA expression (53), there is strong evidence to suggest that the proteasome is impaired in both Alzheimer and Parkinson disease (54, 55). Thus, given that the proteasome appears to be necessary to regulate IL-1 levels, the breakdown in proteasome functionality may be an important contributor to the observed elevation in IL-1 levels in neurodegenerative disease.

Intriguingly, the proteasome is an important therapeutic target in a variety of cancer treatments. One particularly successful therapeutic is the proteasome inhibitor Bortezomib, which has been used to treat a variety of cancers including myeloma, chronic lymphocytic leukemia, prostate cancer, pancreatic cancer, and colon cancer (56). As demonstrated herein, inhibition of the proteasome causes a significant spike in IL-1 levels *in vitro*. If the effect of proteasomal inhibition is mirrored *in vivo*, Bortezomib may also cause an elevation in the IL-1 available for secretion and therefore an increase in inflammation. Although previous evidence appears to negate this hypothesis, suggesting that Bortezomib has an anti-inflammatory effect due to inhibition of the NF-κB pathway (57), there may still be an impact from increased polyubiquitinated IL-1. Thus, the spike in IL-1 observed here *in vitro* may still be of relevance *in vivo* and thus, may still have a considerable impact upon the efficacy of Bortezomib, and potentially any other proteasome inhibitors, as anti-cancer treatments.

In the current investigations, the regulation of IL-1α and IL-1β in DC appear to mirror each other. Although both cytokines serve as proinflammatory cytokines, the exact roles that IL-1α and IL-1β play *in vivo* are distinct. In the initiation of allergic contact dermatitis, for example, IL-1β but not IL-1α appears to be important, whereas in irritant contact dermatitis IL-1α appears to play a more important role (58). Thus, the similarities in IL-1α and IL-1β observed here may not be paralleled in other cell types, other tissue types, or in response to other stimuli. Indeed, in human and murine skin, preformed IL-1α is detected in large amounts, whereas IL-1β is not detectable (41, 42). Although this could be due to differences in transcription, it is plausible that the mechanism of ubiquitination and degradation described herein could be a factor. Specifically, IL-1α degradation may be inhibited in keratinocytes, whereas IL-1β degradation may be functional, effectively resulting in an accumulation of IL-1α.

To conclude, the current investigations show that both IL-1α and IL-1β are tightly regulated by polyubiquitination and proteasomal degradation, adding to the growing body of work that demonstrates that ubiquitination is a key regulator of inflammation and immunity. These findings not only suggest that the mechanism of IL-1 degradation could represent an important therapeutic target, but also highlight that degradation may be pivotal to the maintenance of tissue homeostasis.

## Footnotes

2 The abbreviations used are:

DC: dendritic cells
TLR: Toll-like receptor
NF-κB: nuclear factor κ light chain enhancer of activated B cells
CHX: cycloheximide
BM: bone marrow
WCL: whole cell lysate
DMSO: dimethyl sulfoxide
ANOVA: analysis of variance.
